# Perceived stress, distress, burden, and caregiving preparedness in family caregivers of patients with hematologic malignancies during the first months of treatment

**DOI:** 10.1080/07347332.2026.2629863

**Published:** 2026-05-19

**Authors:** Julia Slack, Sydney Sumrall, Rebecca Hoppe, Marcia A. Winter, Tara A. Albrecht

**Affiliations:** a School of Nursing, Duke University, Durham, North Carolina, USA; b Department of Psychology, Virginia Commonwealth University, Richmond, Virginia, USA; c Department of Psychology, Arizona State University, Tempe, Arizona, USA

**Keywords:** caregiver burden, caregiving preparedness, distress, family caregivers, hematologic malignancies, oncology, quality of life, stress

## Abstract

**Background:**

Hematologic malignancies frequently require intensive treatment, leaving patients to rely on informal caregivers for emotional, logistical, and health care support. This high level of care and support can place an unintended burden on the caregiver. This study examined caregiver wellbeing during the initial treatment period.

**Methods:**

Family caregivers identified by a patient with a hematologic malignancy were enrolled in this longitudinal, descriptive study. Participants answered questionnaires related to distress, stress, caregiver burden, and caregiver preparedness at baseline and six weeks later.

**Results:**

A total of 24 caregivers participated in this study. Caregivers reported moderate to high levels of distress, stress, and caregiver burden across the study period.

**Conclusion:**

Findings suggest that caregivers of patients with hematologic malignancies have high levels of distress and burden during the initial diagnosis and treatment period. Further research and supportive care interventions are warranted to assist the family during this difficult time.

## Introduction

Hematologic malignancies (HMs) are a complex group of cancers that affect the blood and lymph system; they include leukemias, lymphomas, and multiple myeloma. HMs account for approximately 9% of all cancers and as of 2024, there were an estimated 1,698,339 individuals living with or in remission from a HM in the United States.^1^ Despite unprecedented advancements in the treatment of HMs, the regimens are often long, require lengthy hospitalizations, and can result in unexpected complications and emergency room visits.^2–4^ Ultimately, the treatment trajectory results in both physical and psychosocial symptoms that negatively affect the individual’s quality of life.^5–8^ Due to the complexity of care for individuals with HMs, these individuals frequently rely on a close family member or friend to deliver emotional support and care across the treatment trajectory.^9,10^

The family caregiver (FC) is a term commonly used to refer to a close friend or family member who supports the individual going through cancer treatment. The FC typically delivers uncompensated care and assists with transportation, personal care, activities of daily living, and any medical and nursing care that may be needed at home; they also provide emotional support.^11,12^ While treatment is known to be difficult for individuals with HMs, it can be just as challenging for the FCs, resulting in distress and caregiver burden.^13^ However, only recently has research started to explore the burden of care delivered by FCs to individuals with HMs. For example, a recent systematic review exploring the health-related quality of life reported by FC’s of individuals with a HM identified only one longitudinal study out of the 71 included studies, highlighting the gap in our current understanding of FC’s well-being over time.^14^ Understanding well-being and caregiver burden experienced by FCs across the treatment trajectory is essential to delivering quality family-centered care.

## Purpose

The purpose of this study was to explore FCs’ well-being, characterized as stress, distress, and caregiver burden, and their preparedness to provide care, within six weeks of treatment. This period of time typically includes intensive in-hospital treatment for the individual with HM followed by their return home. For FCs, caregiving demands change across this period but are likely to remain intense throughout. Exploring the FCs’ stress, distress, and level of care burden during this period of transition can inform opportunities for future tailored supportive care interventions for the family system.

## Research approach

This longitudinal descriptive study using questionnaires was designed to capture the changes in well-being experienced by FCs of individuals with HMs over the six-week period following diagnosis. Using psychometrically tested instruments FC distress, perceived stress, caregiver burden, and preparedness for caregiving were assessed at two time points: T1/baseline enrollment (within first three-months of diagnosis of HM) and T2/six weeks after enrollment. Capturing the FCs’ experiences at more than one time point allowed for identification of changes and transitions over time within the acute treatment period. The data collected for this study was part of a mixed-methods study which aimed to understand well-being of individuals with HMs and their FCs over the first months of treatment.

### Setting and sample

This study was completed at a National Cancer Institute–designated cancer center in the Mid-Atlantic Region of the United States. Institutional review board approval was obtained. This study included a convenience sample of FCs who were identified by individuals with HMs who had provided informed consent and opted to enroll in the study. The specific inclusion criteria for the FCs was that they needed to be identified by the patient as their primary unpaid caregiver, be 18 years of age or older, and be able to read, write, and speak in English. FCs were excluded if, *via* self-report, they stated that they were experiencing psychiatric, cognitive, or neurological symptoms that prevented the ability to provide consent and/or complete research measures. All FCs provided informed consent prior to data collection. All data collection was completed by a member of the research team who was affiliated with the university but not a member of the participants’ medical or psychosocial team.

## Methods

### Data collection and measures

The FCs completed a battery of measures at two time points, the first was within three months of the HM’s diagnosis, described as the baseline timepoint, and the second was approximately six weeks after enrollment. Questionnaires were collected separately in a private setting, at times convenient for the FC participants. All measures were printed and filled out by FCs using pen and paper. Completed measures were then entered into SPSS v28 by research assistants. FC participants received $25 gift cards at each data collection point (total $50) in appreciation for their time.

The FC participants completed a demographic questionnaire at baseline that included age, gender, race/ethnicity, marital status, education, income, employment status, smoking status, relation of FC to HM patient, living situation, and health status. In addition to demographic data, FCs reported their distress, stress, caregiver burden, and preparedness for caregiving at both timepoints. Data collection occurred August 2019-May 2020. Of note, four FCs that completed the baseline questionnaires did not complete the questionnaires at the six week timepoint, resulting in a 14.3% attrition rate across the study timeframe.

The *Distress Thermometer* (DT) was used to measure the psychological distress of the FC. The DT has been a useful screening tool for both patients and FCs in clinical settings.^15–17^ DT assesses the presence of distress over the past week on a 0–10 numeric scale. Accompanying the DT is a 5-domain (practical, social, emotional, spiritual, and physical) problem list.^16^ The psychometric tests support the validity and reliability of the DT for rapid identification of distress.^17–19^

The *Perceived Stress Scale* was used to assess the degree to which the FCs perceived their situation to be stressful. The Perceived Stress Scale is a 10-item patient rated questionnaire which uses a 5-point Likert scale (0–4 range); it has undergone extensive psychometric testing that supports its reliability and general validity to evaluate perceived stress.^20,21^ For the Perceived Stress Scale, higher scores correspond to worse perceived stress.^20^

The *Caregiver Burden Scale* was used to assess objective burden, subjective demand burden, and subjective stress burden in the FC’s life. The Caregiver Burden Scale has 14-items, with each question’s response ranging from 1 to 5 (a lot less to a lot more). The scale consists of three subscales, objective burden (defined as the perceived infringement or disruption of tangible aspects of a caregiver’s life), subjective demand burden (defined as the extent to which the caregiver perceives care responsibilities to be overly demanding), and subjective stress burden (defined as the emotional impact of caregiving responsibilities on the caregiver). Psychometric testing supports the reliability and validity of this instrument.^22^

The *Preparedness for Caregiving Scale* was used in this study to evaluate the FC’s confidence in delivering physical and emotional care to their patient with a HM. This is an 8-item ordinal scale with scores that range from 0 to 4 with higher scores indicating higher preparedness. This scale has undergone psychometric testing with reported internal consistency scores ranging from .88 to .93.^23^

### Data analysis

Data was downloaded from SPSS into RStudio software program version 2024.04.2. Participants who did not have data for both timepoints were excluded from analysis. Descriptive statistics of demographic data as well as scores from psychometric questionnaires were calculated for all FC participants at baseline and six weeks later. Demographic characteristics were also examined in relation to psychometric data through visualization with bar charts due to the low sample size. The relationship between preparedness for caregiving and distress and burden was ascertained through scatterplot visualizations with linear trend lines. For missing data, if at least 80% of the questions for a questionnaire were answered, a total score was calculated using mean imputation. For questionnaires that had subscales, the mean imputation for missing data was completed based on the mean of the subscale if greater than 50% of the subscale questions were answered. If the participants met the threshold for completed questions but missed greater than 50% of a subscale, a subscale total was not calculated, and the total score was based on mean imputation of the rest of the scale.

After data cleaning, some of the survey data were either dichotomized or trichotomized based on prior research to facilitate interpretation of findings. Specifically, for the Distress Thermometer, a score of 4 or higher (out of 10) was considered moderate/severe distress, as this is the clinical cutoff recommended by the National Comprehensive Cancer Network for cancer patients.^24^ The Perceived Stress Scale total scores were trichotomized to low stress (scores of 0–13), moderate stress (scores of 14–26), and high stress (scores of 27–40) to align with the suggested interpretation for this scale.^20^ Lastly, for the Caregiver Burden Scale the high versus low cutoff was applied according to the questionnaire recommendations:^25^ objective burden was dichotomized at 23; subjective demand burden was dichotomized at 15; subjective stress burden was dichotomized at 13.5.

## Findings

A total of 24 FCs enrolled and completed the questionnaires at both timepoints. The average age of the participants was 56.4 years (SD = 14.4) and the majority were Caucasian (*n* = 19; 79.2%) females (*n* = 17; 70.8%). Most FCs reported their relationship to the patient as either a spouse or significant other (*n* = 15; 62.5%) and lived with the patient receiving treatment for a HM (*n* = 16; 66.7%). FCs also reported some health issues but that they were overall healthy (*n* = 18; 75.0%). Complete demographic information is shown in Table 1.

### Distress

**Figure 1.**
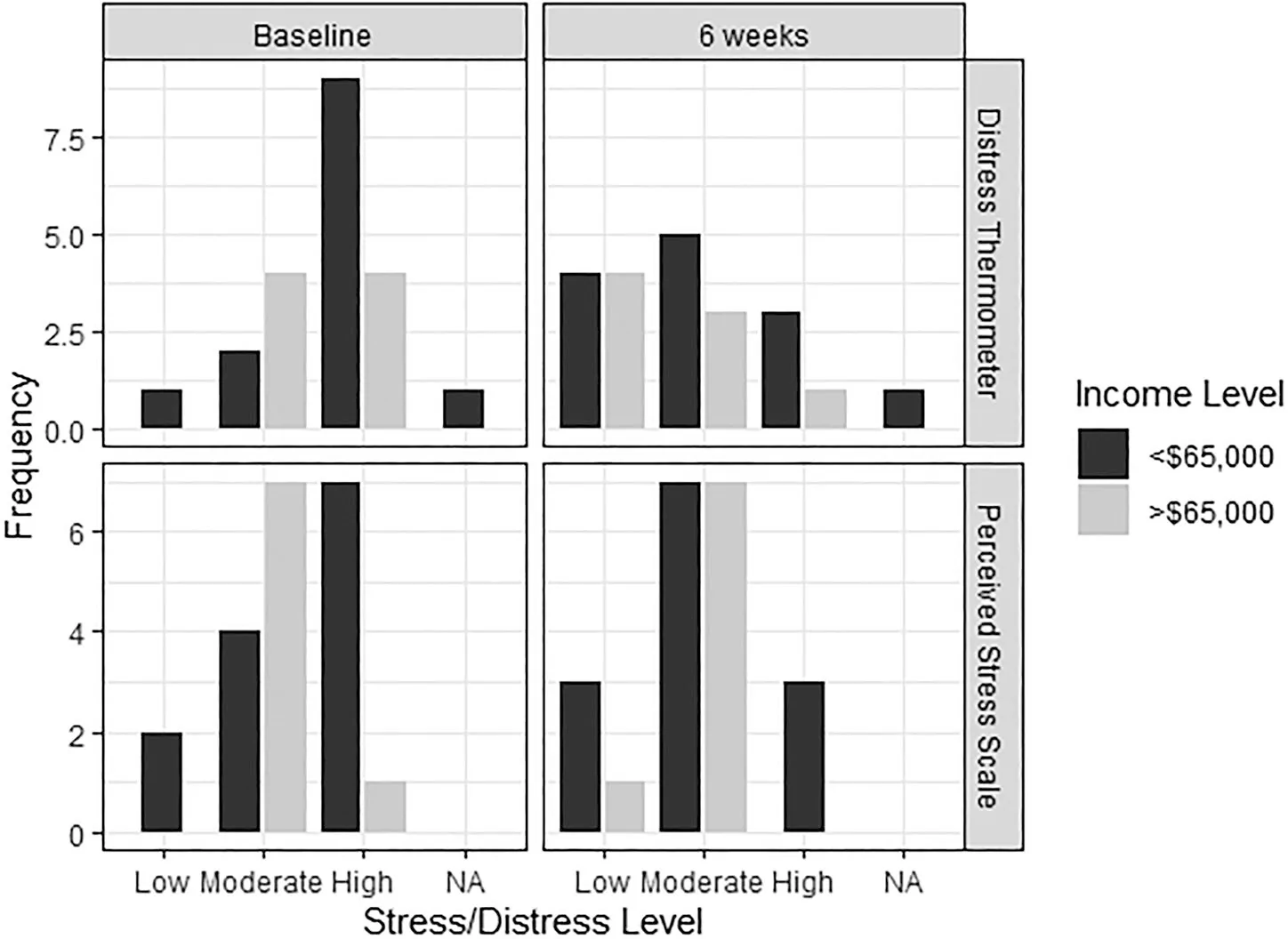
Perceived stress and distress stratified by income level. This bar graph shows the total perceived stress and distress at baseline and 6 weeks based on participant income status stratified at $65,000. Three participants were excluded from this graph due to unknown employment status.

The majority of FCs (*n* = 21; 87.5%) at baseline and 14 FCs (58.3%) at six weeks reported moderate to high levels of distress (Table 2). More specifically, at baseline the mean distress score reported by the FCs was 7.00 (SD = 2.86) and 4.48 (SD = 2.91) at 6 weeks. While overall distress decreased over time, some challenges persisted. Specifically, the mean number on the Emotional subscale (2.0 ± 1.4 vs. 2.0 ± 1.7), Physical subscale (1.8 ± 1.8 vs. 2.3 ± 2.4) and Spiritual Problems subscale (1.9 ± 1.6 vs. 1.9 ± 2.2) which contribute to the overall distress score reported by FCs remained a concern over time. When considering demographic factors, individuals with an income of less than $65,000 had higher distress at baseline compared to individuals with an income over $65,000. Differences in distress between income levels were smaller at the second timepoint but still present (Figure 1).

### Perceived stress

Perceived stress levels of FCs in this study were high at both baseline and six weeks. At baseline 10 (41.7%) FCs documented high stress levels, 11 FCs (45.8%) reported medium stress levels, and only 3 (12.5%) felt they experienced low stress; the mean stress level was 24.4 (SD = 9.92). Stress levels remained elevated at six weeks as 19 FCs (79.2%) still reported medium or high levels of distress, and the overall mean score was 20.0 (SD = 8.16).

### Caregiver burden

**Figure 2.**
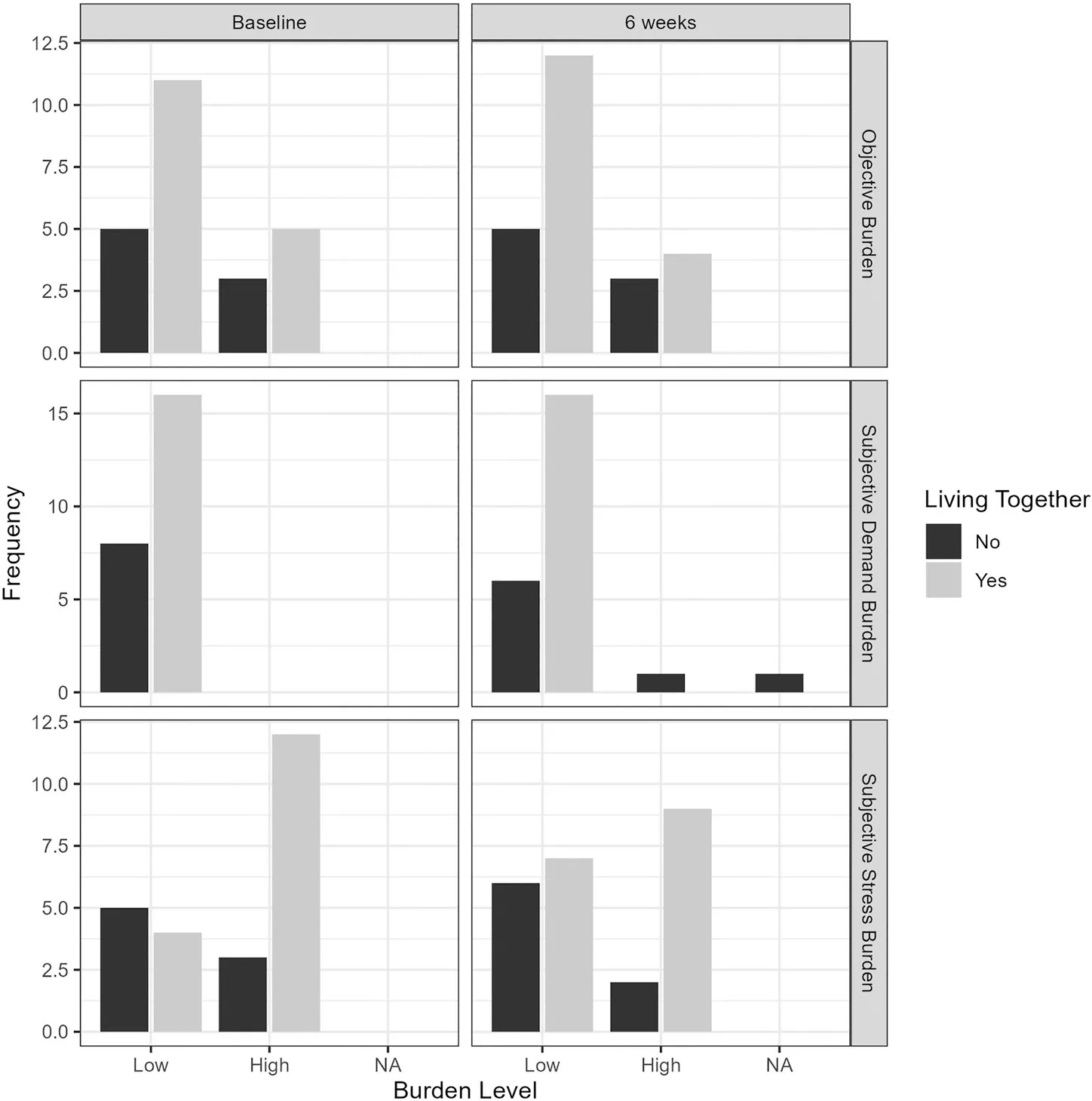
caregiver burden stratified by living status. This bar graph shows the caregiver burden subscales at baseline and 6 weeks stratified by whether the family caregiver was living with the patient with a hematologic malignancy or not.

The mean Caregiver Burden score reported by FCs remained relatively consistent over time: 44.3 ± 6.37 at baseline to 44.8 ± 6.9 at six weeks, indicating caregiver burden did not improve over time. Of the three subscales, the subjective stress burden was the most elevated subscale for participants with 15 FCs (62.5%) stating high burden at baseline and only slightly fewer FCs (*n* = 11; 45.8%) reporting high subjective stress burden by week six. Objective burden remained relatively consistent over time as 8 (33.3%) and 7 (29.2%) FCs reported high objective burden at baseline and six weeks, respectively. No FCs experienced a high subjective demand burden at baseline and only one person had a high subjective demand burden at six weeks. When caregiver burden was stratified by living status, those FCs who were living with their patient reported a higher caregiver burden compared to those who did not live with the patient with HM. Specifically, the FCs that lived with their patient more frequently reported a high level of subjective stress burden (Figure 2). Neither subjective demand burden nor objective burden appeared to differ by living status.

### Preparedness for caregiving

**Figure 3.**
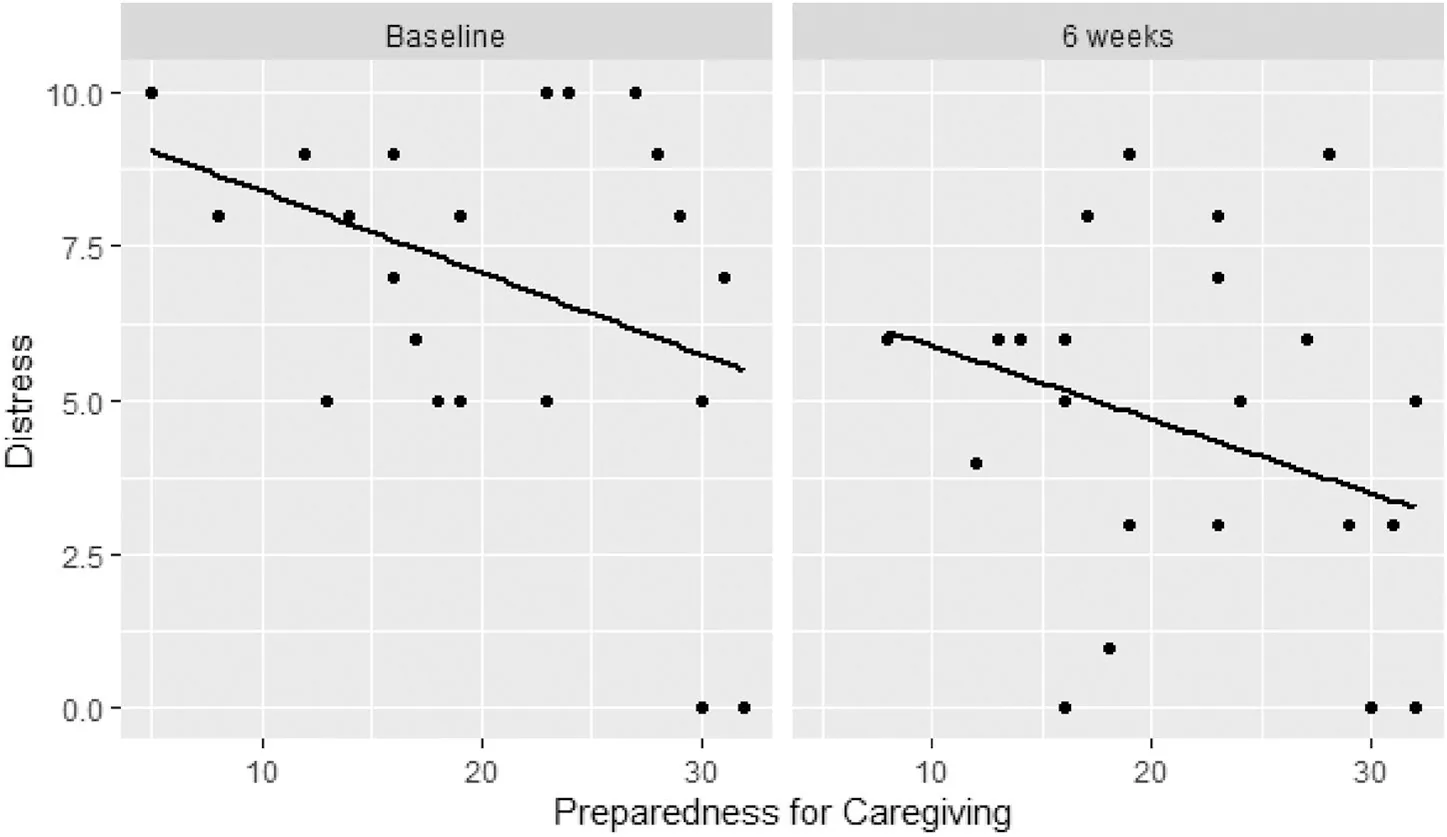
The relationship between preparedness for caregiving and distress. This scatterplot shows the relationship between family caregiver reported preparedness for caregiving and distress at baseline and 6 weeks with a straight trend line.

**Figure 4.**
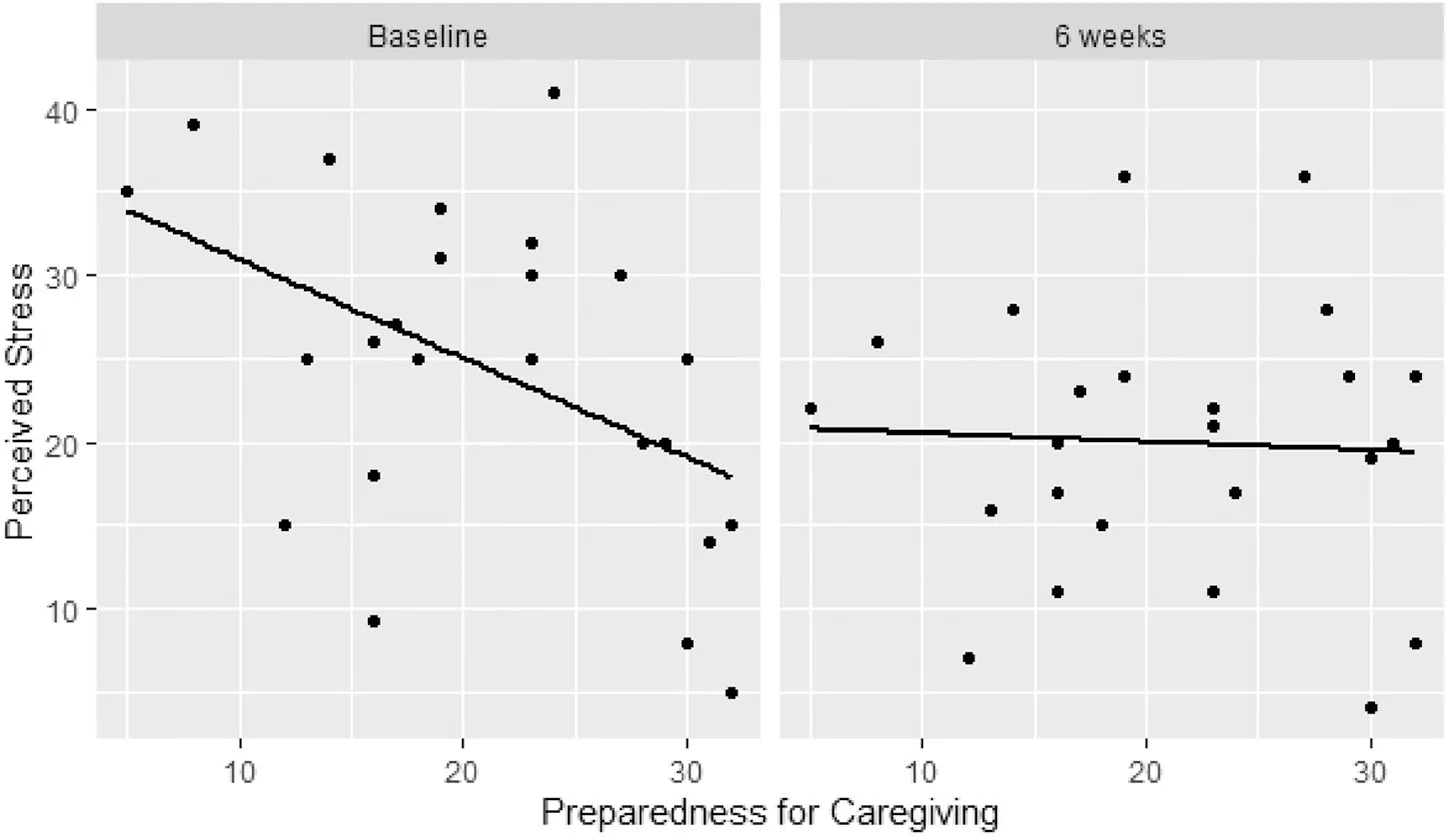
The relationship between preparedness for caregiving and perceived stress. This scatterplot shows the relationship between family caregiver reported preparedness for caregiving and perceived stress at baseline and 6 weeks with a straight trend line.

**Figure 5.**
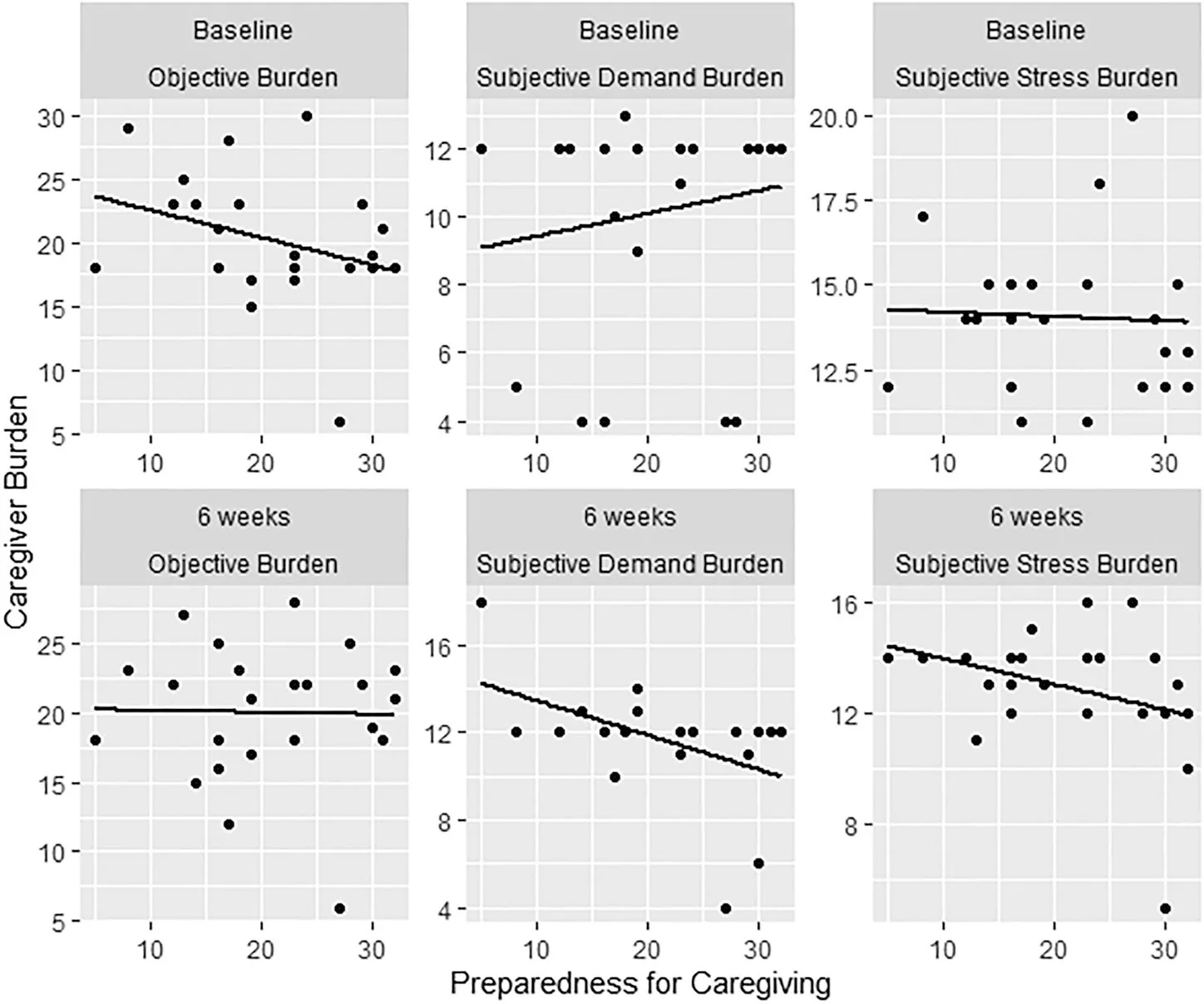
The relationship between preparedness for caregiving and the caregiver burden subscales. This scatterplot shows the relationship between family caregiver reported preparedness for caregiving and the caregiver burden subscales at baseline and 6 weeks with a straight trend line

The mean preparedness for caregiving scores reported by the FCs increased from 21.04 (SD = 7.80) at baseline to 23.13 (SD = 7.52) at six weeks. A higher level of reported preparedness for caregiving was associated with less distress at both timepoints based on scatterplot visualizations (Figure 3). The relationship of preparedness for caregiving with perceived stress changed over time. Preparedness for caregiving was inversely linked to perceived stress at baseline, but no strong association between preparedness and perceived stress was seen at six weeks (Figure 4). The relationship between caregiver preparedness and the caregiver burden subscales also varied over time. A higher reported preparedness for caregiving was related to a higher subjective demand burden at baseline. Yet, higher caregiver preparedness was correlated with less subjective demand burden at 6 wk. For objective burden and subjective stress burden, a trend between higher reported preparedness for caregiving and decreased burden was only strongly evident based on the scatterplot visualization at one of the two timepoints for both subscales, baseline for objective burden and six weeks for subjective stress burden, with the other showing minimal relationship (Figure 5).

## Discussion

This study explored FCs’ wellbeing caring for individuals with a HM over the initial treatment period. Our findings indicated that on average, the FCs experience high levels of distress, stress, and caregiver burden across the entire study period. The subjective stress burden was particularly concerning as approximately half of the FCs reported a high burden at both timepoints. Additionally, this study found that the FCs who lived with their patient reported a worse subjective stress burden based on the Caregiver Burden Scale than the FCs who did not live with their patient. This relationship between caregiver burden and living situation aligns with a previous integrative review on the experience of FCs of individuals with cancer, which found that caregiver burden was higher amongst FCs living with the individual under their care compared to those FCs who did not live with the individual requiring informal care assistance.^26^ The review also found that older age and male gender were associated with increased caregiver burden and while these correlations were not clearly found in this study, the lack of evident associations may be due to the small sample size.^26^

In addition to the reported high levels of caregiver burden, distress was also reported to be high for the majority of the FCs across the study period. The DT subscale for physical problems that are contributing to the level of distress, which relates to physical symptoms such as fatigue and pain, was particularly notable as the FCs indicated modestly worse distress in this category across time. A subset of FCs also reported worse overall distress as well as perceived stress at six weeks compared to baseline. These findings align with our study team’s previous qualitative work which illustrated that FCs of individuals with HMs experience complex care needs, including but not limited needing improved interventions to better address distress, stress, and burden.^13^ Similarly, a literature review that focused on the FCs of individuals with HMs also found that FCs reported high levels of distress and caregiver burden during treatment. However, only one of the included studies in the review was longitudinal.^14^ Thus, our longitudinal study which demonstrates that distress remains persistent for FCs across treatment timepoints is noteworthy. However, this study only examined the initial treatment period and as treatment for individuals with HMs extends well beyond six weeks, future longitudinal studies should consider extending assessment over a longer timeframe to provide a clearer understanding of FC wellbeing over the entire treatment trajectory. Also of note, a past study which used principal component analysis to examine both the Distress Thermometer and the Caregiver Burden Scale for FCs of individuals with lung cancer found that high subjective stress burden was associated with high distress thermometer scores; the study also found that the FCs experienced high levels of both subjective stress burden and distress.^27^ Thus, while our study did not examine the interconnectedness of these concepts, future research should explore potential causal links between subjective stress burden and distress to better inform tailored interventions and improve the FC experience.

While FCs in this study reported high distress, stress, and caregiver burden, a higher preparedness for caregiving was associated with improved well-being for some of the measures. Specifically, higher preparedness for caregiving reported by the FC’s corresponded with decreased distress at both timepoints. Preparedness for caregiving was also correlated with perceived stress, objective burden, and subjective stress burden during at least one of the two timepoints in this study. Interestingly, a higher reported level of preparedness for caregiving was related to a higher subjective demand burden at baseline which was unexpected and contradictory to findings at six weeks which found that higher preparedness was associated with less burden. One potential reason for this counterintuitive relationship at baseline could be that the FCs who reported more preparedness for caregiving also felt that the tasks their patients asked them to perform (that they had not prepared for) were excessive or overly demanding, regardless of the true nature of the request. Conversely, the less prepared FCs had less of an idea of what to expect so viewed all caregiving tasks equally and were thus not burdened by unexpected requests. The inverse relationship between caregiver preparedness and distress, stress, and burden has been previously documented in the literature for various caregiver populations.^28–30^ Thus, future research should explore whether interventions focused on improving the FC’s preparedness for caregiving to individuals with HMs will lead to improved FC well-being.

### Clinical and research implications

This study found that FCs of individuals with HMs experience high levels of distress, stress, and caregiver burden which remain prevalent throughout the first months of treatment. Future research should build upon these findings to investigate longitudinal associations between psychosocial impacts of treatment and control for variables that may impact these associations, such as income and living situation. Additionally, as demonstrated in a systematic review and meta-analysis of 24 studies, existing early palliative care interventions have shown limited improvements to FC well-being.^31^ Future studies should investigate how palliative and other interventions can mitigate distress, stress, and caregiver burden in FCs of individuals with HMs, particularly during diagnosis and early treatment. For example, online peer support groups and virtual counseling have been found to lower care burden of FCs of individuals with leukemia.^32,33^ Further exploration of psychosocial interventions is warranted to better understand the utility of these interventions to manage FC stress, distress, and caregiving burden during the initial treatment period.

In addition to highlighting the need for future research on both FC well-being during the entire treatment trajectory as well as the effectiveness of psychosocial interventions to manage FC stress, distress, and caregiver burden, this study also has important clinical implications. Primarily, health care providers for individuals with HMs should assess the well-being of their patient’s FC during the initial treatment period. As evidenced by this study, FCs of individuals with HMs experience high and sustained stress, distress, and caregiver burden; thus, connecting with the FCs to discuss their well-being and making referrals to supportive care interventions is critical. Health care providers can also help increase FCs’ preparedness, and therefore their well-being, by providing thorough information regarding caregiving expectations and responsibilities throughout the patient’s treatment trajectory.

### Limitations

This study had several limitations, one of which is the lack of sociodemographic diversity in the sample. As most of the FCs who participated in this study were relatively healthy White/European American individuals caring for their spouse/significant other, the findings might not be generalizable to other populations. Another limitation of the study is that a few of the FCs completed the second questionnaire during the beginning of the COVID-19 pandemic, which could have resulted in the FCs experiencing higher levels – or qualitatively different types – of distress and stress due to the pandemic. Finally, the small sample size of the mixed-method parent study renders this quantitative analysis descriptive and exploratory, and did not allow for the inclusion of covariates in analyses. However, despite these limitations, this study provides valuable insight into the experience of family caregivers caring for individuals with HMs.

## Conclusion

Findings from this study suggest that FCs of individuals with HMs experience high levels of distress, stress, and caregiver burden that remain persistent from baseline to six weeks into treatment. Thus, further evaluation and support of the FC by the clinical team should be considered. Given that specialty palliative care provides support to not only the patient but also to the family system, professionals should consider initiating early palliative care referrals for individuals with HMs and their FCs to improve management of their complex care needs and foster better patient and FC health. Future studies should explore how to mitigate distress, stress, and caregiver burden in FCs of individuals with HMs through the use of tailored interventions. Specifically, studies should explore the link between preparedness for caregiving and overall FC well-being to determine if interventions centered on increasing preparedness would lead to improved FC well-being. Ultimately, further longitudinal study of FC experiences can inform novel interventions to support quality family-centered care.

## Figures and Tables

**Table 1.** Sample demographics.

Variable	M (SD) or *N* (%)

Age	56.4 (14.4)
Gender	
Male	7 (29.2%)
Female	17 (70.8%)
Race/ethnicity	
African American	3 (12.5%)
White/European American	19 (79.2%)
Latino/a	0 (0.0%)
Other/missing	2 (8.4%)
Income	
<65,000	13 (54.2%)
>65,000	8 (33.3%)
Missing	3 (12.5%)
Marital status	
Single/Divorced	5 (20.8%)
Married	17 (70.8%)
Widowed	2 (8.3%)
Employment status	
Full-time	8 (33.3%)
Unemployed	3 (12.5%)
Retired	10 (41.7%)
Part-time	2 (8.3%)
Missing	1 (4.2%)
Education	
Some College or Less	12 (50.0%)
College/Trade School Graduate +	12 (50.0%)
Current smoking status	
No	21 (87.5%)
Yes	2 (8.3%)
Missing	1 (4.2%)
Health description	
I am very healthy	5 (20.8%)
I have some health issues but am overall healthy	18 (75.0%)
I have health issues that limit my ability to do things	1 (4.2%)
Living situation	
Does not live with patient	8 (33.3%)
Lives with patient	16 (66.7%)
Relationship to patient	
Spouse/significant other	15 (62.5%)
Parent	3 (12.5%)
Child	3 (12.5%)
Other	3 (12.5%)
Patient diagnosis	
AML	12 (50.0%)
ALL	6 (25.0%)
APML	1 (4.2%)
NHL	3 (12.5%)
Other/Missing	2 (8.3%)

Abbreviations: M = mean; SD = standard deviation; AML = acute myeloid leukemia; ALL = acute lymphoblastic leukemia; APML = acute promyelocytic leukemia; NHL = non-Hodgkins’s lymphoma.

**Table 2.** Family caregiver experience.

Variable	Timepoint 1 M (SD) or *N* (%)	Timepoint 2 M (SD) or *N* (%)

DT Total Score	7.00 (±2.86)	4.48 (±2.91)
High	15 (62.5%)	5 (20.8%)
Moderate	6 (25.0%)	9 (37.5%)
Low	2 (8.3%)	9 (37.5%)
Missing	1 (4.2%)	1 (4.2%)
PSS Total Score	24.4 (±9.92)	20.0 (±8.16)
High	10 (41.7%)	4 (16.7%)
Moderate	11 (45.8%)	15 (62.5%)
Low	3 (12.5%)	5 (20.8%)
Caregiver Burden Scale Total Score	44.3 (±6.37)	44.8 (±6.93)
*Objective burden*	20.13 (±5.02)	20.00 (±4.83)
High	8 (33.3%)	7 (29.2%)
Low	16 (66.7%)	17 (70.8%)
*Subjective demand burden*	10. 17 (±3.23)	11. 65 (±2.57)
High	0 (0.0%)	1 (4.2%)
Low	24 (100.0%)	22 (91.7%)
Missing	0 (0.0%)	1 (4.2%)
*Subjective stress burden*	14.04 (±2.16)	12.92 (±2.21)
High	15 (62.5%)	11 (45.8%)
Low	9 (37.5%)	13 (54.2%)
Preparedness for Caregiving	21.04 (±7.80)	23.13 (±7.52)

M = mean; SD = standard deviation; DT = Distress Thermometer; PSS = Perceived Stress Scale.
